# Speech acoustic indices for differential diagnosis between Parkinson’s disease, multiple system atrophy and progressive supranuclear palsy

**DOI:** 10.1038/s41531-022-00389-6

**Published:** 2022-10-27

**Authors:** Khalid Daoudi, Biswajit Das, Tereza Tykalova, Jiri Klempir, Jan Rusz

**Affiliations:** 1 INRIA Bordeaux Sud-Ouest (GeoStat team), Talence, France; 2 Department of Circuit Theory. Faculty of Electrical Engineering, Czech Technical University in Prague, Prague, Czech Republic; 3 Department of Neurology and Center of Clinical Neuroscience, First Faculty of Medicine, Charles University and General University Hospital, Prague, Czech Republic

**Keywords:** Parkinson's disease, Diagnostic markers

## Abstract

While speech disorder represents an early and prominent clinical feature of atypical parkinsonian syndromes such as multiple system atrophy (MSA) and progressive supranuclear palsy (PSP), little is known about the sensitivity of speech assessment as a potential diagnostic tool. Speech samples were acquired from 215 subjects, including 25 MSA, 20 PSP, 20 Parkinson’s disease participants, and 150 healthy controls. The accurate differential diagnosis of dysarthria subtypes was based on the quantitative acoustic analysis of 26 speech dimensions related to phonation, articulation, prosody, and timing. A semi-supervised weighting-based approach was then applied to find the best feature combinations for separation between PSP and MSA. Dysarthria was perceptible in all PSP and MSA patients and consisted of a combination of hypokinetic, spastic, and ataxic components. Speech features related to respiratory dysfunction, imprecise consonants, monopitch, slow speaking rate, and subharmonics contributed to worse performance in PSP than MSA, whereas phonatory instability, timing abnormalities, and articulatory decay were more distinctive for MSA compared to PSP. The combination of distinct speech patterns via objective acoustic evaluation was able to discriminate between PSP and MSA with very high accuracy of up to 89% as well as between PSP/MSA and PD with up to 87%. Dysarthria severity in MSA/PSP was related to overall disease severity. Speech disorders reflect the differing underlying pathophysiology of tauopathy in PSP and *α*-synucleinopathy in MSA. Vocal assessment may provide a low-cost alternative screening method to existing subjective clinical assessment and imaging diagnostic approaches.

## Introduction

Idiopathic Parkinson’s disease (PD) is a neurological disorder which affects 1.6 % of the population over 65 years and which is featured by the progressive loss of dopaminergicneurons in the substantia nigra pars compacta. It has been shown that concentrations of dopamine are significantly reduced prior to the apparition of motor deficits^1,2^. The cardinal signs of PD, usually referred to as parkinsonism, include postural instability, bradykinesia, resting tremor and muscular rigidity. Other neurodegenerative diseases that go beyond the signs and symptoms of parkinsonism are known as atypical parkinsonian syndromes (APS). Multiple system atrophy (MSA) and progressive supranuclear palsy (PSP) are subgroups of APS, with a prevalence around 30–40 per 100,000 among the popoulation older than 65 years^3^. Clinical features of PSP include supranuclear gaze palsy, axial rigidity, bradykinesia, frequent falls, cognitive decline and communication disorders^4,5^, reflecting widespread neurodegeneration involving the midbrain as well as the hypothalamic nucleus, globus pallidus, pons, striatum, superior cerebellar peduncle and cerebellar dentate nucleus^4^. Conversely, MSA is characterized by various combinations of parkinsonian, autonomic and cerebellar features^6^, corresponding to degeneration of striatum, substantia nigra, middle cerebellar peduncle, cerebellum, inferior olivary nucleus and pons^7^. APS differ from PD by a poor response to levodopa and more rapid progression of the disease, resulting in a shorter life expectancy^8,9^.

The majority of PD and APS patients manifest similar clinical features which might render very challenging a correct differential diagnosis^10^. There exists clinical criteria for the diagnosis of "probable” and "possible” MSA and PSP, based on clinical or/and imaging features, but the definite MSA and PSP diagnosis requires postmortem confirmation by a neuropathological examination^7^. Currently, several imaging techniques such as MRI, positron emission tomography, diffusion tensor imaging, single-photon emission computed tomography and transcranial sonography can be used to assess various parkinsonian syndromes^11^. In particular, automatic image-based classification based on metabolic patterns is highly accurate in differentiating between PD, MSA and PSP patients at early disease stages, with more than 84% sensitivity and 94% specificity^12^. However, metabolic imaging is burdened by the invasive use of radiopharmaceuticals, whilst financial costs and technical demands may limit the use of other imaging methods.

It is now well established that dysarthria, a class of motor speech impairments resulting from neurological disorders, is an early clinical feature of PD and APS^13^. Due to the dysfunction of the basal ganglia, most of PD patients manifest hypokinetic dysarthria which is characterized by monoloudness, monopitch, variable rate, reduced stress, harsh voice quality, imprecise articulation, inappropriate silence and speech dysfluencies^14,15^. Conversely, MSA and PSP patients typically manifest mixed dysarthria with a combination of ataxia, hypokinesia and spasticity as a result of more widespread neuronal atrophy^16–18^. Indeed, previous studies^16,17^ which investigated 44 PSP and 46 MSA patients using perceptual speech and oral motor analysis have reported mixed dysarthria with combinations of all ataxic, hypokinetic and spastic components in two-thirds of the patients. Spastic components were mostly present in PSP patients, while hypokinetic components followed by ataxic components were predominant in MSA patients^16,17^. Dysarthria can manifest in all the levels of speech production^19^: respiration, articulation, phonation, timing and prosody (and nasality to a lower extent).

During the last decades, PD speech analysis has gained an increasing interest. However, the majority of research have focused on distinguishing between PD and healthy subjects with the motivation to use speech assessment as a supporting method for early PD diagnosis. While this may be interesting from a fundamental perspective or for monitoring purposes, it has actually a limited impact in clinical diagnosis. Indeed, early PD diagnosis cannot be claimed, as often done, when APS dysarthria is not taken into account. Moreover, the clinical diagnosis often even neglects the possibility of an APS. The resulting speech corpora may thus be noisy in the sense that some patients diagnosed as PD may actually be APS. Such studies may claim at best features/methods which correlate with a diagnosis of *parkinsonism* (which groups PD and APS), acknowledging that parkinsonism does not require speech analysis to be correctly diagnosed.

On the other hand, there exists only few studies on comparison/discrimination between PD and APS or between APS subgroups^13,20–31^. In this work we focused on this challenging problem by using, first, an assessment of all basic subsystems of connected speech: timing, prosody, articulation, phonation and respiration. Then, based on the findings, we proposed an assessment based dysarthria subtypes: hypokinetic, ataxic and spastic.

## Results

### Univariate statistical analysis

**Fig. 1:**
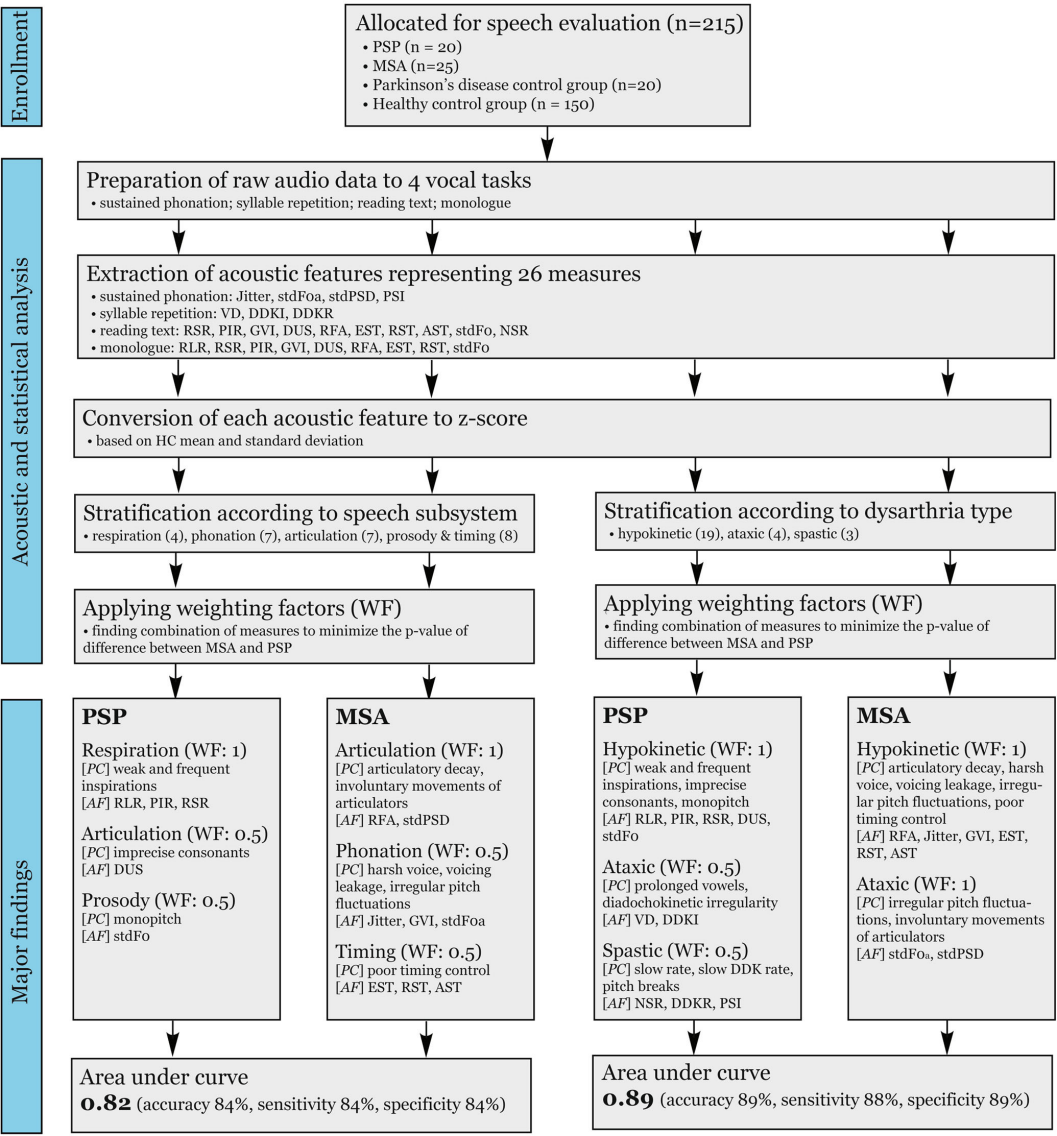
Scheme chart depicting the methodology and major findings. PC hypothesized perceptual correlates, AF acoustic features, WF weighting factor, RLR relative loudness of respiration, PIR pause intervals per respiration, RSR rate of speech respiration, DUS duration of stop consonants, stdF0 standard deviation of fundamental frequency, RFA resonant frequency attenuation, stdPSD standard deviation of the power spectral density, GVI gaping in-between voiced intervals, stdF0a pitch fluctuations, EST entropy of speech timing, RST rate of speech timing, AST acceleration of speech timing, VD vowel duration, DDKI diadochokinetic instability, NSR net speech rate, DDKR diadochokinetic rate, PSI proportion of sub-harmonic intervals, PSP progressive supranuclear palsy, MSA multiple system atrophy.

The overview of methodology and major findings can be seen in Fig. 1 (Supplementary Audio S1–S4). Univariate statistical analysis of the initial acoustic features, described in Table 7, is shown in Table 1. Only 2 features yielded individually a significant group difference between PSP and MSA, *stdF*0_*a*_ and *RFA*_*m*_ (*p* ~ 0.01). *DUS*_*m*_, *stdPSD* and *RFA*_*t*_ approached significant group difference with *p* ~ 0.06. However, classification accuracy was poor using these features individually.

**Table 1:** Statistical difference between groups.

Feature	HC	PD	PSP	MSA	HC vs PD	HC vs PSP	HC vs MSA	PSP vs MSA
	Mean/SD (Range)	Mean/SD (Range)	Mean/SD (Range)	Mean/SD (Range)	*p* value	*p* value	*p* value	*p* value
Hypokinetic								
* RLR*_*m*_	−24.66/3.80 (−33.09–14.77)	−25.47/3.46 (−30.63–16.72)	−28.35/5.11 (−37.89–18.49)	−27.21/3.47 (−36.25–22.10)	0.28	**0.002**	**0.005**	0.26
* RSR*_*m*_	17.08/4.36 (8.02–33.13)	16.22/4.58 (9.35–26.99)	21.68/5.51 (9.18–34.54)	18.57/4.12 (9.82–24.74)	0.36	**0.0004**	0.06	0.06
* RSR*_*t*_	17.03/4.00 (7.41–27.93)	18.11/4.10 (9.23–26.08)	21.48/6.17 (11.68–32.81)	18.63/5.26 (7.09–27.43)	0.38	**0.004**	0.12	0.18
* PIR*_*m*_	5.13/1.76 (2.00–13.00)	5.47/2.11 (2.00–12.00)	2.76/1.52 (1.00–6.00)	3.18/1.47 (1.00–6.50)	0.53	**0**	**0**	0.26
* PIR*_*t*_	6.43/1.78 (3.00–13.75)	6.44/1.52 (4.00–10.00)	4.24/2.58 (1.50–11.00)	4.55/1.85 (2.25–10.00)	0.78	**0**	**0**	0.33
* jitter*_*a*_	0.48/0.24 (0.20–2.58)	0.47/0.23 (0.23–1.30)	0.65/0.35 (0.27–1.42)	0.71/0.37 (0.32–1.79)	0.34	0.2	**0.0006**	0.25
* GVI*_*m*_	43.53/11.22 (11.45–72.14)	40.58/14.28 (15.32–66.33)	24.97/11.42 (8.66–44.47)	24.06/9.06 (7.94–40.72)	0.41	**0**	**0**	0.87
* GVI*_*t*_	56.50/11.00 (24.90–78.96)	55.66/13.54 (25.91–74.56)	39.78/16.46 (13.84–65.76)	40.20/12.22 (18.64–66.77)	0.96	**0**	**0**	0.93
* DUS*_*m*_	25.24/9.89 (13.38–85.38)	29.91/16.60 (17.88–74.12)	48.78/17.40 (22.38–85.38)	39.20/13.61 (17.88–67.38)	0.46	**0**	**0**	0.06
* DUS*_*t*_	22.42/7.43 (11.12–66.25)	27.38/14.48 (15.62–73.00)	37.30/13.49 (20.12–62.88)	33.72/16.36 (15.62–92.12)	0.28	**0**	**0.00001**	0.23
* RFA*_*m*_	9.42/1.40 (6.32–13.35)	8.22/1.37 (5.61–10.79)	9.95/1.63 (7.70–14.02)	8.64/1.26 (6.54–10.98)	**0.001**	0.25	**0.013**	**0.013**
* RFA*_*t*_	10.68/1.54 (7.40–15.76)	9.61/1.22 (7.55–11.18)	11.24/1.78 (8.93–14.97)	10.19/1.25 (7.54–12.57)	**0.007**	0.25	0.18	0.06
* EST*_*m*_	1.55/0.01 (1.50–1.58)	1.55/0.01 (1.53–1.56)	1.54/0.01 (1.51–1.56)	1.53/0.02 (1.47–1.56)	0.11	**0.012**	**0.0003**	0.62
* EST*_*t*_	1.55/0.01 (1.50–1.57)	1.55/0.01 (1.53–1.57)	1.54/0.02 (1.49–1.57)	1.54/0.01 (1.52–1.56)	0.58	**0.002**	**0.0007**	0.45
* RST*_*m*_	364.11/60.58 (170.97–511.27)	356.40/82.75 (198.44–524.13)	241.96/86.53 (128.34–410.73)	247.24/76.25 (127.60–411.75)	0.45	**0.000001**	**0**	0.73
* RST*_*t*_	433.34/52.65 (270.35–595.46)	434.83/79.03 (304.48–653.87)	315.13/113.02 (148.11–498.11)	338.58/69.85 (206.45–474.38)	0.91	**0.00001**	**0**	0.51
* AST*_*t*_	19.37/13.88 (−12.30–56.94)	21.49/14.26 (−8.74–47.81)	8.74/10.47 (−8.23–30.16)	7.15/15.05 (−15.46–40.13)	0.35	**0.002**	**0.0001**	0.49
* stdF*0_*m*_	2.00/0.73 (0.84–6.26)	1.47/0.40 (0.81–2.29)	1.72/0.46 (1.09–2.64)	1.48/0.34 (0.88–2.35)	**0.0002**	0.1	**0.00003**	0.16
* stdF*0_*t*_	2.51/0.75 (0.93–5.80)	1.72/0.60 (0.97–3.16)	1.28/0.27 (0.81–1.91)	1.38/0.51 (0.76–3.23)	**0.000004**	**0**	**0**	0.95
Ataxic								
* stdF*0_*a*_	0.35/0.24 (0.11–2.00)	0.36/0.28 (0.13–1.38)	0.50/0.30 (0.16–1.08)	0.69/0.25 (0.28–1.14)	0.85	**0.02**	**0**	**0.01**
* stdPSD*_*a*_	2.22/0.39 (1.37–3.48)	2.12/0.37 (1.41–3.04)	2.22/0.34 (1.65–2.91)	2.56/0.42 (1.85–3.96)	0.36	0.97	**0.0004**	0.06
* VD*_*s*_	49.63/9.82 (28.16–86.72)	50.38/9.45 (37.88–74.56)	95.08/52.53 (44.34–221.91)	73.96/21.67 (38.03–116.88)	0.89	**0.000002**	**0.000001**	0.4
* DDKI*_*s*_	22.50/11.82 (8.37–86.02)	26.90/12.05 (10.02–47.57)	85.75/55.11 (11.04–215.26)	63.85/29.47 (22.85–119.87)	0.1	**0**	**0**	0.29
Spastic								
* PSI*_*a*_	4.96/10.06 (0.00–71.64)	6.24/9.35 (0.00–35.70)	11.51/15.06 (0.00–55.02)	10.69/13.02 (0.00–55.87)	0.4	0.2	**0.003**	0.55
* DDKR*_*s*_	6.45/0.72 (3.97–8.51)	6.61/0.78 (5.26–8.19)	4.93/1.44 (2.31–7.78)	5.35/0.94 (3.52–7.23)	0.35	**0.000007**	**0.000001**	0.18
* NSR*_*t*_	2.41/0.27 (1.67–3.17)	2.43/0.28 (1.98–2.99)	2.04/0.60 (1.01–3.61)	2.14/0.39 (1.24–2.82)	0.94	**0.0003**	**0.0009**	0.4

Bold numbers indicate significant differences (*p* < 0.05). Only features which yielded a significant difference between at least two groups are reported. Subscripts *a*, *s*, *m* and *t* indicate that the feature is computed using sustained /a/, syllable repetition, monologue and reading passage, respectively.

**Fig. 2:**
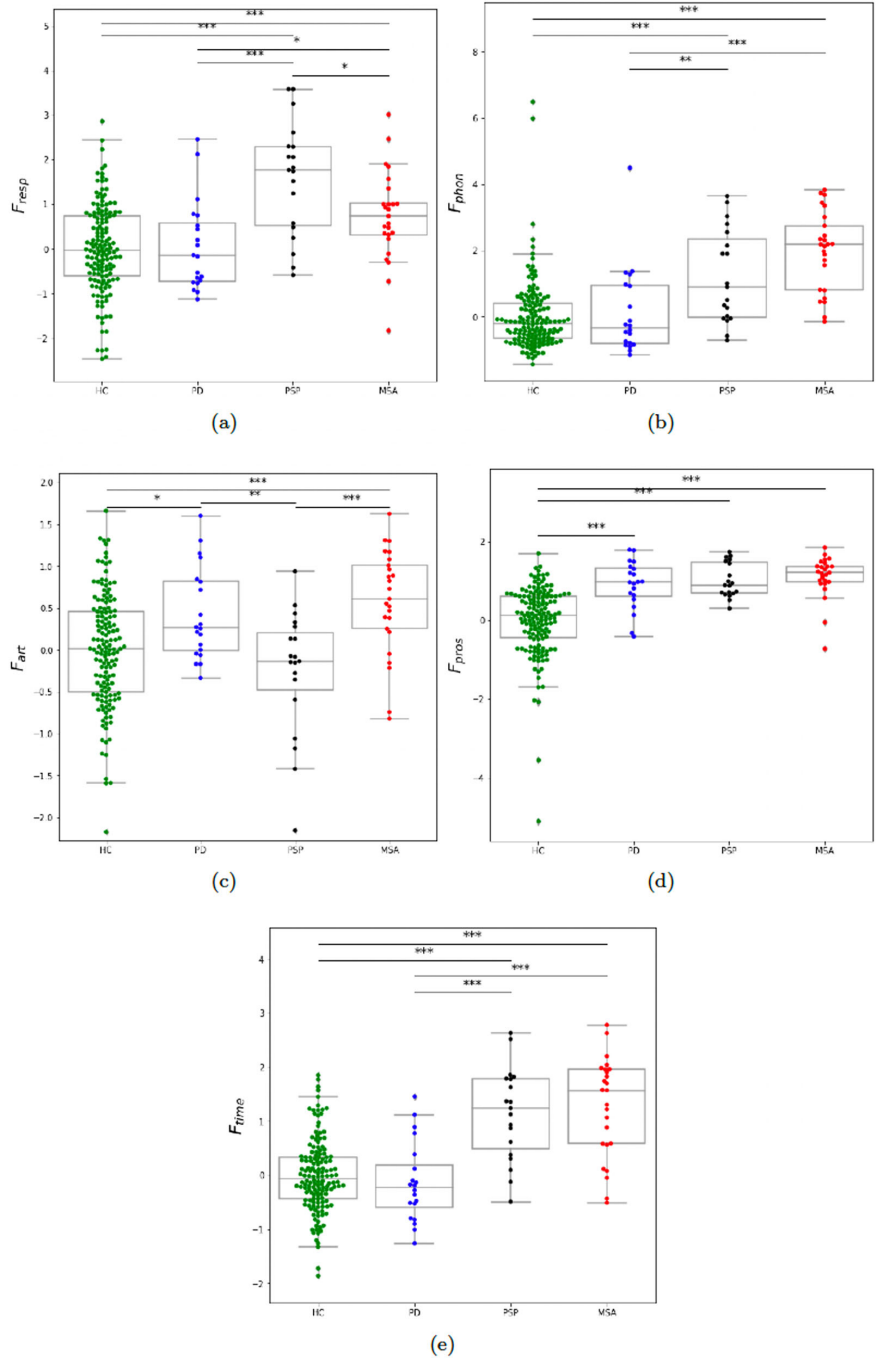
Boxpolts of the distribution across groups of subsystem features. **a** *F*_*resp*_ = respiration feature; **b** *F*_*phon*_ = phonation feature; **c** *F*_*art*_ = articulation feature; **d** *F*_*pros*_ = prosodic feature; **e** *F*_*time*_ = timing feature. HC healthy controls, PD Parkinson’s disease, MSA multiple system atrophy, PSP progressive supranuclear palsy. Statistically significant differences between groups: *p < 0.05, **p < 0.01, ***p < 0.001.

The result of the statistical analysis of the designed subsystem features (see the Methods section) is illustrated in Fig. 2. The comparison between groups using *F*_*resp*_ is shown in Fig. 2a. Using this feature, a respiration deficit is reflected in both MSA and PSP, the latter showed however a greater severity compared to MSA (*p* < 0.05). As shown in Fig. 2b, we found that both MSA and PSP develop a significant phonation impairment as measured by *F*_*phon*_ (*p* < 0.00001 when compared to HC). However, this impairment was more pronounced for MSA (*p* = 0.08 for MSA vs. PSP). Using the articulation feature *F*_*art*_, we did not find a statistical significance between PSP and HC as shown in Fig. 2c, suggesting that *F*_*art*_ does not capture a particular articulation impairment in PSP (this does not mean that PSP do not develop articulation impairment in general). On the other hand, we found that it reflects a significant articulation impairment in MSA (*p* < 0.0001 compared to both PSP and HC). We did not find group difference between PSP and MSA using *F*_*pros*_, as illustrated in Fig. 2d. However, as could be expected, we found that monopitch (measured by *F*_*pros*_) was prominent not only for PSP and MSA but also for PD, (*p* < 0.00001 compared to HC). We did not find group difference between PSP and MSA using *F*_*time*_, as illustrated in 2(e). However, timing deficit (measured by *F*_*time*_) showed a severe impairment for PSP and MSA, as compared to HC (*p* < 0.00001) and PD (*p* < 0.0001).

**Fig. 3:**
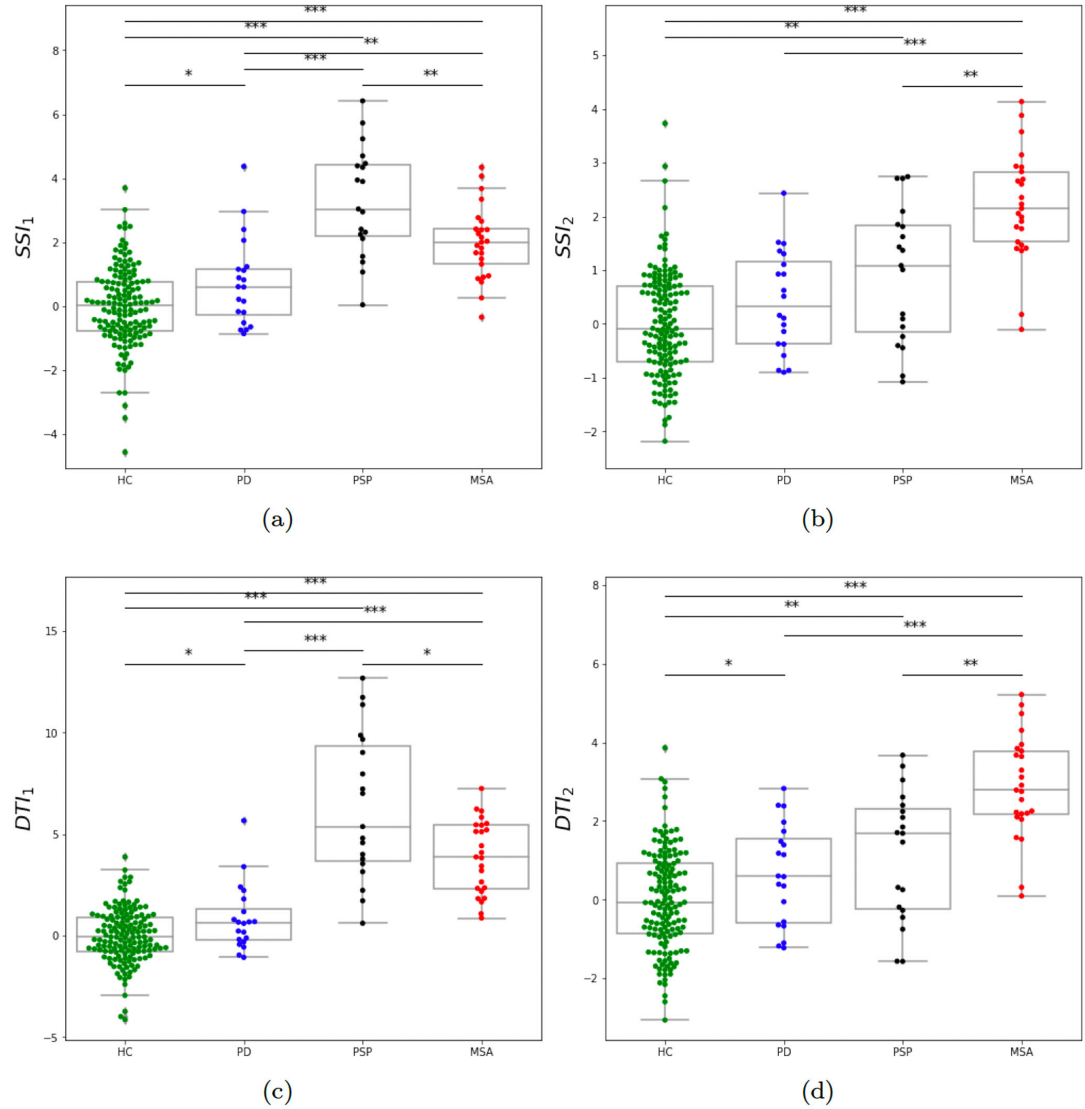
Boxpolts of the distribution across groups of subsystem and dysarthria type indices. **a** *SSI*_1_ = first subsystem index; **b** *SSI*_2_ = second subsystem index; **c** *DTI*_1_ = first dysarthria type index; **d** *DTI*_2_ = second dysarthria type index. HC healthy controls, PD Parkinson’s disease, MSA multiple system atrophy, PSP progressive supranuclear palsy. Statistically significant differences between groups: *p < 0.05, **p < 0.01, ***p < 0.001.

The result of the statistical analysis of the designed indices (see the Methods section) is illustrated in Fig. 3. Using the index *SSI*_1_, the impairment is more predominant in PSP as shown in Fig. 3a. Moreover, the *SSI*_1_ yields a mutual statistically significant difference between all pairs of groups. We recall here that we did not use neither PD nor HC data in the design process. This indicates that *SSI*_1_ has a strong potential in the discrimination between all groups. Using the index *SSI*_2_, the impairment is more predominant in MSA as shown in Fig. 3b. However, it does not reflect a particular impairment of PD. Using the index *DTI*_1_, the impairment is more predominant in PSP as shown in Fig. 3c. Moreover, as *SSI*_1_, *DTI*_1_ yields a mutual statistically significant difference between all pairs of groups. This indicates that *DTI*_1_ has also a strong potential in the discrimination between all groups. Using the index *DTI*_2_, the impairment is more predominant in MSA as shown in Fig. 3d. Moreover, *DTI*_2_ yields a mutual statistically significant difference between all pairs of groups, except between PD and PSP.

### Bivariate classification analysis

**Fig. 4:**
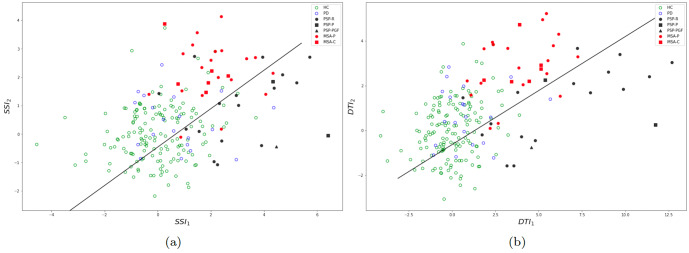
Two-dimensional projection of all subjects over the indices. **a** Using the subsystem indices *SSI*_1_ and *SSI*_2_, **b** using the dysarthria type indices *DTI*_1_ and *DTI*_2_. The black line is the logistic regression boundary for the classification between PSP and MSA using all data.

Figure 4a (resp. b) displays the 2–dimensional distribution of the subsystem indices *SSI*_1_ and *SSI*_2_ (resp. *DTI*_1_ and *DTI*_2_). One can visually observe that both representations achieved a good mutual separation between PD, MSA and PSP groups. Table 2 shows the scores of classification between PSP and MSA using all indices. Individual indices did not give a good classification performance. However, the composite indices, *CSSI* and *CDTI*, yielded a high classification performance, thanks to the orthogonality incorporated in their design. *CSSI* gave very good classification scores (84%) which is already significantly higher than accuracies reported in the literature. *CDTI* yielded an even higher performance (>88%) showing that including prior knowledge on predominant dysarthria types in PSP and MSA does indeed improve the discriminative power.

**Table 2:** Classification accuracy between PSP and MSA.

Feature	AUC	Accuracy (%)	Specificity (%)	Sensitivity (%)
*SSI*_1_	0.68	72.7	80.0	63.1
*SSI*_2_	0.72	70.4	92.0	42.1
**CSSI**	**0.82**	**84.0**	**84.0**	**84.2**
*DTI*_1_	0.65	75.0	96.0	47.3
*DTI*_2_	0.74	70.4	92.0	42.1
**CDTI**	**0.89**	**88.6**	**88.0**	**89.4**

Bold numbers indicate the subsystem and dysarthria type indices which yielded the highest classification scores.

Table 3 shows the scores of classification between PD and MSA. Individual indices yielded a relatively low specificity, except *DTI*_2_. However, the composite indices, *CSSI* and *CDTI*, yielded a high classification performance. Table 4 shows the scores of classification between PD and PSP. Here, *DTI*_1_ alone yield a high classification performance. We recall that *PD* data was not used in the design of the features and indices. These results can be thus seen as an additional posterior validation of the pertinence of our approach.

**Table 3:** Classification accuracy between PD and MSA.

Feature	AUC	Accuracy (%)	Specificity (%)	Sensitivity (%)
*SSI*_1_	0.73	71.1	65.0	76.0
*SSI*_2_	0.88	84.4	75.0	92.0
**CSSI**	**0.88**	**86.6**	**85.0**	**88.0**
*DTI*_1_	0.88	84.4	75.0	92.0
*DTI*_2_	0.87	82.2	80.0	84.0
**CDTI**	**0.91**	**86.6**	**85.0**	**88.0**

Bold numbers indicate the subsystem and dysarthria type indices which yielded the highest classification scores.

**Table 4:** Classification accuracy between PD and PSP.

Feature	AUC	Accuracy (%)	Specificity (%)	Sensitivity (%)
*SSI*_1_	0.85	79.5	80.0	78.9
*SSI*_2_	0.51	56.4	95.0	15.8
**CSSI**	**0.85**	**82.0**	**75.0**	**89.4**
**DTI**_1_	**0.92**	**87.1**	**90.0**	**84.2**
*DTI*_2_	0.46	51.2	100	5.2
*CDTI*	0.90	84.6	85.0	84.2

Bold numbers indicate the subsystem and dysarthria type indices which yielded the highest classification scores.

### Relation between speech and motor manifestations

The correlations between the speech indices and bradykinesia/rigidity, bulbar, cerebellar and overall NNIPPS scores in the APS (PSP + MSA) group are shown in Table 5. The overall NNIPPS score was related to the indices *SSI*_2_ and *DTI*_2_ (*r* = 0.5, *p* < 0.01). These two indices also showed strong correlation with the bradykinesia/rigidity and cerebellar NNIPPS subscores (*r* ~ 0.5, *p* < 0.01). They were not however correlated to the bulbar subscore. No correlation were detected between the NNIPPS (sub)scores and the indices *SSI*_1_ and *DTI*_1_.

**Table 5:** Correlations between speech and clinical motor indices.

Speech index/NNIPPS score and subscores	Bradykinesia/Rigidity	Bulbar	Cerebellar	NNIPPS score
*SSI*_1_	−0.05	0.18	−0.2	−0.06
*SSI*_2_	0.52**	0.18	0.48**	0.5**
*DTI*_1_	0.02	0.25	−0.16	0.15
*DTI*_2_	0.53**	0.16	0.49**	0.51**

Statistically significant differences between groups: *p < 0.05, **p < 0.01.

## Discussion

Our findings indicate that speech disorders reflect the differing underlying pathophysiology of tauopathy in PSP and *α*-synucleinopathy in MSA. The combination of distinct speech patterns via objective acoustic evaluation was able to discriminate between PSP and MSA with a very high accuracy of up to 88.6%, though the difference was not perceptually identifiable using the UPDRS III speech item. This is in accordance with systematic perceptual assessment, which was not able to distinguish between the speech of PSP and MSA^32^. To the best of our knowledge, this is the best accuracy reported in the literature concerning speech-based discrimination between two APS with diverging pathophysiology. Although PD data was not used in training, our approach also separated both PSP and MSA from PD with an accuracy of up to 87%, reflecting the fact that dysarthria severity was considerably higher in APS than PD. This finding is consistent with previous studies showing that, at mid/late stages, APS can be distinguished from PD by speech deterioration severity using both perceptual and acoustic analysis^13,32^. The greater severity of dysarthria in APS was reflected likely because early-stage PD patients manifest pure hypokinetic dysarthria^33^, whereas APS patients had dysarthria with different combinations of hypokinesia, ataxia, or spasticity^16,17^.

Speech features related to respiratory dysfunction, imprecise consonants, and monopitch, assessed via the *SSI*_1_ dimension, contributed to worse performance in PSP than MSA. The influence of respiratory dysfunction on speech was not yet systematically studied. However, it is well known that patients with PSP have profound impairment of voluntary respiratory control^34^. In addition, PSP patients had significantly more frequent respiratory infections and respiratory-related deaths when compared to PD patients^35^. Voiceless consonant abnormalities in PSP have also been reported and associated with perceptual severity of dysarthria^29^. Although a similar extent of monotone speech was found in both PSP and MSA, the distinguishing accuracy of pitch variability might be attributed to wider performance variability in MSA due to more frequent ataxic components causing excessive pitch fluctuations^17,18^, as compared to the typical occurrence of hypokinetic and spastic elements of dysarthria in PSP^16,36^. The relevance of spastic speech components in PSP was further underlined by findings of slow speaking rate and subharmonics that contributed to discrimination accuracy between PSP and MSA via the *DTI*_1_ dimension. Both slow speaking rate and subharmonics are considered to be the core features encountered in spastic dysarthria as a result of more widespread neuronal atrophy^37,38^. In particular, the relation between slow articulation rate and bilateral white and gray matter volume loss was observed in patients with progressive spastic dysarthria^39^ and patients with multiple sclerosis with predominant spastic-ataxic dysarthria^40^. Surprisingly, ataxic features reflecting higher diadochokinetic irregularity and prolonged phonemes were affected on average more in PSP than MSA. While the longer phonemes may reflect a slower diadochokinetic rate, the slightly higher diadochokinetic irregularity in PSP might be simply caused by a higher number of patients with severe dysarthria (40% in PSP vs. 32% in MSA), which appear to be the main significant factor influencing oral diadochokinetic performance^41,42^. Another possible explanation is that cerebellar characteristics in PSP may be related to the high concentration of tauprotein deposits in the brainstem where the cerebello-thalamo-cortical and cortico-ponto-cerebellar pathways pass^43^.

The phonation and timing abnormalities and articulatory decay generally contributed to worse performance in MSA compared to PSP via both *SSI*_2_ and *DTI*_2_ indices. Our findings demonstrate overall poorer voice control in MSA, typically manifested as the strained-strangled voice that may give the perceptual impression of quivery-croaky strained speech with increased pitch^18^. This observation is generally in agreement with a recent study showing that 93% of patients with MSA manifested laryngeal dysfunction during an endoscopic task, in contrast with only 1.8% of patients with PD^44^. The slightly worse performance in articulatory decay in MSA than PSP might be attributed to more distorted vowels that are more common in ataxic dysarthria^38,45^. Timing abnormalities were relatively non-specific and affected to a similar extent in both PSP and MSA; however, some timing abnormalities in individual MSA patients might still contribute to discrimination accuracy together with phonatory and articulatory dysfunction.

In patients with APS, an overlap of individual speech features among dysarthria subtypes can be expected^38^, which makes the correct recognition of a specific dysarthria subtype challenging. Although we strived to separate hypokinetic, ataxic and spastic components of dysarthria as much as possible to eliminate most of this overlap, some dysarthric manifestations may still originate from different neuronal dysfunctions and more than anticipated. For instance, subharmonics may arise due to the involvement of the corticobulbar pathways but also cerebellum and basal ganglia. In particular, variation in speech severity within a dysarthria subtype may explain as much variance in acoustic or perceptual data as variation across dysarthria type^46^. This assumption was further confirmed by the relationships revealed only between *SSI*_2_ and *DTI*_2_ dysarthria indexes and overall disease severity by NNIPPS, with no specific correlations observed between hypokinetic, ataxic and spastic speech dimensions and bradykinesia/rigidity, ataxia, and bulbar motor manifestations, respectively.

Our findings are based on sophisticated acoustic analyses, which might limit their application to movement disorders specialists or general neurologists. On the other hand, the majority of applied acoustic features correspond to the main perceptual dimensions encountered in dysarthria of parkinsonism (Fig. 1)^16,17^, and therefore we believe experience clinicians can still profit from the knowledge of distinctive speech characteristics revealed in the present study. In addition, the fully-automated Dysarthria Analyzer used in this study remains under development, but the free beta version is already available^47^. The most of investigated speech features can also be analyzed using widely-used, freely-available Praat software, although hand-labeling or additional user control of the analysis is required for some features. Last but not least, the detailed protocol on speech tasks and speech metrics used in this study has already been published within a recent guideline for speech recording and acoustic analyses in dysarthrias of movement disorders^48^.

One potential limitation of the present study is that we did not differentiate between speech in the various subtypes of PSP and MSA due to the limited opportunity to recruit a larger number of participants via a single center. According to the previous research, it appears that different subtypes of disease have no substantial effect on individual acoustic features^21^, although more perceptual ataxic abnormalities can be observed in MSA cerebellar subtype compared to parkinsonian subtype^31^. In our study, patients with MSA cerebellar subtype had mostly all components of dysarthria affected and thus did not principally differed from type of dysarthria observed in MSA parkinsonian subtype. Another limitation is that we did not perform additional testing including cerebrospinal fluid biomarkers and radionuclide scanning to improve the accuracy of clinical diagnosis. Also, our results are based on participants recruited from a single center and thus might not be universal among races. Future research is warranted to confirm and extend our findings via international collaborative effort leading to involvement of different languages and various racial groups. Finally, we did not investigate loudness of speech, which is an important distinguishing feature of hypokinetic dysarthria, because of the need for precise microphone calibration to obtain reliable estimates via acoustic analysis.

In conclusion, our findings highlight that detailed speech analysis can be used as a potential diagnostic screening tool to distinguish between PSP, MSA, and PD. Therefore, this study can become the basis for future multicenter studies in parkinsonism with speech testing. Future studies based on the earlier stages of the disease and potentially accompanied by longitudinal assessment should further elaborate and extend our findings and show the sensitivity of speech investigation in the differentiation between APS. Since the speech impairment in PD appears to be a progressive biomarker that reflects dopaminergic treatment response^33^ and progression in APS is more rapid and less responsible to L-dopa therapy^3^, longitudinal in-home assessment over a short period could substantially improve our reported sensitivity of speech-based evaluation. As a result, a vocal assessment may provide a no-cost alternative screening method to existing clinical and imaging diagnostic approaches.

## Methods

### Participants

For the present study, From 2011 to 2018, we recruited a total of 65 patients via a single center, 25 with a diagnosis of probable MSA (15 men and 10 women), 20 with a diagnosis of probable PSP (13 men and 7 women) and 20 with a diagnosis of idiopathic PD (13 men and 7 women). A specialist of movement disorders established the diagnoses of all patients according to the consensus diagnostic criteria for MSA^7^, the Movement Disorder society diagnostic criteria for PSP^49^ and the Movement Disorder Society clinical diagnostic criteria for PD^50^. The MSA group consisted in 19 patients diagnosed with MSA-parkinsonian (MSA-P) subtype and 6 patients with MSA-cerebellar (MSA-C) subtype while the PSP group was composed of 17 patients diagnosed with PSP-Richardson (PSP-R) syndrome, 2 with PSP-parkinsonism (PSP-P) and 1 with PSP-pure akinesia with gait freezing (PSP-PGF). At the time of examination, each treated MSA and PSP patient was on stable medication, for at least 4 weeks, consisting of various doses of levodopa alone or combined with different dopamine agonizts and/or amantadine. PD patients were examined immediately after the diagnosis and before the initiation of dopaminergic treatment. No PD subject manifested dyskinesias at the time of the examination. Disease duration was estimated based on the self-reported manifestation of the first motor symptoms. Each PSP and MSA patient underwent neurological examination including scoring according to the Neuroprotection and Natural History in Parkinson Plus Syndromes (NNIPPS) scale^51^, while PD patients were rated by the Movement Disorder Society - Unified Parkinson’s Disease Rating Scale (MDS-UPDRS) motor subscore. None of the patients reported a history of speech-language disorders unrelated to potential neurologic disorder manifestations. No statistically significant differences were found between MSA and PSP groups for medication doses, disease duration, cognitive status, speech or motor severity (Mann–Whitney *U* test: *p* = 0.11–0.59). Dysarthria presence, severity and type were evaluated based on the auditory-perceptual judgment of a speech-language specialist experienced in movement disorders using audio recordings of vowel phonation, /pa/-/ta/-/ka/ syllable repetition, and monologue following the perceptual criteria described in ref. ^14^. Patient clinical and demographic characteristics are summarized in Table 6. The control group consisted of 150 healthy subjects of comparable gender distribution (95 men and 55 women; 63% male gender) as well as age (mean age 65.5, SD 7.1, range 45–83 years, *p* = 0.08 between controls and PSP and MSA). No control subject reported a history of neurological disorders or other disorders that may affect speech, language or hearing. A significant difference in age distribution was found among PSP, MSA, PD and controls (ANOVA: *p* = 0.002) mainly due to the slightly younger PD group compared to MSA (*p* = 0.01) as well as PSP (*p* = 0.01). All patients and controls were Czech native speakers, and none manifested a cognitive or depressive deficit that would bias with the recording procedure. The study was approved by the Ethics Committee of the General University Hospital in Prague, Czech Republic (approval number 34/18 Grant AZV VES 2019 1.LF UK) and have therefore been performed in accordance with the ethical standards laid down in the 1964 Declaration of Helsinki and its later amendments. All participants provided written, informed consent to the neurological examination and recording procedure.

**Table 6:** Patient clinical and demographic characteristics.

	PSP (*n* = 20)	MSA (*n* = 25)	PD (*n* = 20)	*p* value
General				
Male gender (%)	13 (65)	15 (60)	13 (65)	0.92
Age (years)	67.1 ± 6.2 (54–84)	62.5 ± 6.7 (45–72)	59.1 ± 13.6 (37–81)	0.07
Disease duration^b^ (years)	4.0 ± 1.5 (2–7)	3.9 ± 1.6 (2–7.5)	3.0 ± 1.7 (0.3–6.7)	0.07
Levodopa equivalent (mg/day)	545 ± 501 (0–1500)	371 ± 457 (0–1500)	0	<0.001^a^
Amantadine (mg/day)	155 ± 167 (0–500)	82 ± 120 (0–400)	0	<0.001^a^
NNIPPS total	69.7 ± 25.4 (19–116)	79.0 ± 23.5 (35–123)	–	0.29
NNIPPS mental function	8.4 ± 4.6 (0–17)	6.8 ± 3.9 (0–14)	–	0.35
MDS-UPDRS III total	–	–	30.3 ± 11.0 (10–53)	–
MDS-UPDRS III speech item	2.0 ± 0.7 (1–3)	1.8 ± 0.6 (1–3)	0.6 ± 0.5 (0–1)	<0.001^a^
Dysarthria severity^c^				
None (%)	0	0	2 (10)	0.10
Mild (%)	3 (15) PSP-R, 1 (5) PSP-P	5 (20) MSA-P, 1 (4) MSA-C	16 (80)	<0.001^a^
Moderate (%)	7 (35) PSP-R, 1 (5) PSP-PGF	7 (28) MSA-P, 4 (16) MSA-C	2 (10)	0.04^a^
Severe (%)	7 (35) PSP-R, 1 (5) PSP-P	7 (28) MSA-P, 1 (4) MSA-C	0	0.007^a^
Dysarthria type^c^				
Hypokinetic (%)	1 (5) PSP-R	4 (16) MSA-P	18 (100)	<0.001^a^
Hypokinetic - spastic (%)	7 (35) PSP-R, 1 (5) PSP-P, 1 (5) PSP-PGF	4 (16) MSA-P, 1 (4) MSA-C	0	0.002^a^
Hypokinetic - ataxic (%)	2 (10) PSP-R	7 (28) MSA-P	0	0.02^a^
Hypokinetic - spastic - ataxic (%)	6 (30) PSP-R	3 (12) MSA-P, 4 (16) MSA-C	0	0.03^a^
Ataxic (%)	1 (5) PSP-P	1 (4) MSA-P, 1 (4) MSA-C	0	0.44
Ataxic - spastic (%)	1 (5) PSP-R	0	0	0.32

Data are mean ± SD (range) or number (percent) including *p* values between PSP, MSA and PD analyzed using Kruskall–Wallis test or number (%) analyzed using Chi-square test.

*PSP* progressive supranuclear palsy, *MSA* multiple system atrophy, *PD* Parkinson’s disease, *MSA-P* multiple system atrophy parkinsonian subtype, *MSA-C* multiple system atrophy cerebellar subtype, *PSP-R* progressive supranuclear palsy Richardson syndrome, *PSP-P* progressive supranuclear palsy parkinsonism, *PSP-PGF* progressive supranuclear palsy pure akinesia with gait freezing, *NNIPPS* Natural History and Neuroprotection on Parkinson plus syndromes-Parkinson plus scale, *MDS-UPDRS* Movement Disorder Society - Unified Parkinson’s disease Scale.

^a^No significant differences between PSP and MSA groups.

^b^Based on the self-reported occurrence of first motor symptoms.

^c^Based on perceptual criteria outlined by Darley et al. (1969)^14^.

### Speech recording

Speech was recorded in a quiet room with a low ambient noise level using a head-mounted condenser microphone (Beyerdynamic Opus 55, Heilbronn, Germany) placed ~5 cm from the subject’s mouth. Speech signals were sampled at 48 kHz with 16-bit resolution. Each subject was recorded during a single session with a speech specialist. All participants performed 4 vocal tasks of (i) sustained phonation of the vowel /a/ per one breath for as long and steadily as possible, (ii) fast /pa/-/ta/-/ka/ syllable repetition at least seven times per one breath, (iii) reading a short paragraph of a standard text composed of 80 words and (iv) monologue on a self-chosen theme during ~90 s. These speech tasks were chosen because they provide comprehensive information for the objective interpretation and description of motor speech disorders^48^. Sustained phonation, fast syllable repetition and text reading were carried out twice per session by each subject.

### Initial acoustic speech features

We performed a quantitative acoustic vocal assessment of 26 distinct speech dimensions related to hypokinetic (19), ataxic (4) and spastic (3) dysarthria with subsystems consisting of respiration (4), phonation (7), articulation (7), prosody and speech timing (8). Acoustic analysis was preferred because it provides objective, sensitive and quantifiable information for the precise assessment of speech performance from very early stages of PD^52^. Considering hypokinetic dimensions and respiratory features, we obtained relative loudness respiration (RLR), rate of speech respiration (RSR) and pause intervals per respiration (PIR) via reading passage/monologue. To assess hypokinetic dimensions and phonatory features, we calculated jitter, shimmer and harmonics-to-noise ratio (HNR) via sustained phonation and gaping in-between voiced intervals (GVI) via reading passage/monologue. To examine hypokinetic dimensions and articulatory features, we assessed duration of stop consonants (DUS), resonance frequency attenuation (RFA) via reading passage/monologue, as well as voice onset time (VOT) via syllable repetition. To explore hypokinetic dimensions and timing features, we calculated duration of pause intervals (DPI), entropy of speech timing (EST), rate of speech timing (RST) and acceleration of speech timing (AST) via reading passage/monologue. To investigate hypokinetic features and prosody, we assessed standard deviation of fundamental frequency (stdF0) via reading passage/monologue. Subsequently, ataxic features of pitch fluctuation (stdF0; phonation) and standard deviation of the power spectral density (stdPSD; articulation) were examined via sustained phonation which represent phonation and articulation deficits, respectively, while vowel duration (VD; timing) and diadochokinetic instability (DDKI; timing) were assessed via syllable repetition. Finally, three spastic features of proportion of sub-harmonic intervals (PSI; phonation), diadochokinetic rate (DDKR; articulation) and net speech rate (NSR; timing) were calculated via sustained phonation, syllable repetition and reading passage, respectively. The list of initial speech features we used is given in Table 7. Comprehensive algorithmic details on individual acoustics measures have been reported previously^53^. Also, the accuracy of algorithms for the identification of glottal cycles, temporal intervals, and pitch sequence has been thoroughly tested in previous studies^22,53,54^.

**Table 7:** List of the initial acoustic features: Mlg = Monologue task and Txt = Reading passage task.

Deviant speech dimension (unit)	Vocal task	Subsystem	Definition	Description
Hypokinetic				
RLR: Relative loudness of respiration (db)	Mlg	Respiration	Computed as difference between median loudness of respiration and median loudness of speech.	Decreased or increased inspiratory effort.
RSR: Rate of speech respiration (ms)	Mlg+Txt	Respiration	Estimated as median duration between respirations.	Increased rate of speech respiration. Inefficient respiration due to weakness of respiratory muscles or restricted range of movements.
PIR: pause intervals per respiration (respiration/mn)	Mlg+Txt	Respiration	Measured as mean number of pauses between respirations.	Decreased ability to control respiratory airflow.
Jitter (%)	Sustained phonation	Phonation	Short-time fundamental frequency perturbation	Harsh voice
GVI: Gaping in-between voiced intervals (pause/mn)	Mlg+Txt	Phonation	Clear pauses (i.e. pauses in-between voiced speech)	Decreased ability of vocal folds to stop voicing by adduction.
DUS: Duration of stop consonants (ms)	Mlg+Txt	Articulation	Articulatory aspects were quantified for unvoiced fricatives and stop consonants independently	The stability of supra-laryngeal movements and explosion about its preciseness
RFA: Resonance frequency attenuation (db)	Mlg+Txt	Articulation	RFA is measured as the difference between the second formant region and the minima of the local valley.	Acoustic resonances are less prominent due to articulatory imperfections such as mumbling.
EST: Entropy of speech timing (-)	Mlg+Txt	Timing	EST is defined as Shannon entropy applied on incidences of speech intervals.	Limited control and coordination of articulators yield ordered and predictable speech.
RST: Rate of speech timing (intervals/mn)	Mlg+Txt	Timing	RST is defined as the total number of voiced, unvoiced, and pause intervals was accumulated during the time course.	Reduced range of movement disturb the timing and coordination of speech subsystems.
AST: Acceleration of speech timing (‰/*mn*^2^)	Txt	Timing	Computed as the difference of RST between two overlapping halftimes divided by total time.	The tendency to accelerate speech rate.
stdF0: Standard deviation of fundamental frequency (semitone)	Mlg+Txt	Prosody	The stdF0 is computed as the standard deviation of detected modal F0 in semitones estimated via the median absolute deviation.	Monotone voice, lacking normal pitch and inflection changes
Ataxic				
stdF0_*a*_: Pitch fluctuation (semitone)	Sustained phonation	Phonation	The stdF0 is estimated as the standard deviation of the F0.	Irregular or timing deficits of vocal folds vibration.
stdPSD: Standard deviation of the power spectral density (db)	Sustained phonation	Articulation	The stdPSD is calculated as the mean value of the standard deviations in band power	The severity of involuntary movements of articulators.
VD: Vowel duration (ms)	Syllable repetition	Timing	The VD is estimated as the mean duration of detected voiced intervals.	The slowness of repetitive movements with excessive vocal emphasis.
DDKI: Diadochokinetic instability (ms)	Syllable repetition	Timing	The DDKI is estimated as the standard deviation of the measured durations between consecutive voice onsets.	Irregular or timing deficits of repetitive movements.
Spastic				
PSI: Proportion of sub-harmonic intervals (%)	Sustained phonation	Phonation	PSI is calculated as the ratio between the total duration of sub-harmonic intervals per total duration of voicing.	Changes in the mass or control of vocal folds.
DDKR: Diadochokinetic rate (syllables/s)	Syllable repetition	Articulation	The DDKR is estimated as the inversion of the median duration between consecutive voice onsets.	Abnormally slow motion rate of articulators
NSR: Net speech rate (syllables/s)	Txt	Timing	The total number of syllables was divided by the total duration of speech.	Slowness of individual movements of articulators.

### Design of acoustic indices by subsystem tasks

We first carried out a univariate statistical analysis of the features presented above. This analysis showed that individual features do not lead to an acceptable discrimination performance. We thus considered linear combination of different features as described in the following.

Individual features were first converted to the z-score using the HC mean and standard deviation. For acoustic features in which lower raw scores was associated with greater dysarthria, the z-score was reversed. Thus, higher z-scores were indicating more speech impairment. We then followed a semi-supervised approach to find feature combinations. As our ultimate goal was to design indices that would not be overfitted to our dataset and would allow easy reproducibility, we restricted feature combinations to averaging. Moreover, the designed features were chosen carefully to minimize the potential overlap between subsystem dysarthria in order to achieve a certain orthogonality between indices. For each subsystem task, to find the best combination of measures for separation between groups (HC vs. MSA, HC vs. PSP and MSA vs. PSP), an exhaustive search over all averages over the acoustic features of that task was performed. The separation performance was measured in term of minimizing the *p* value of difference between MSA and PSP groups (threshold of significance was set at *p* < 0.05). Using this search, a combination satisfying this criterion was found only in the articulation subsystem. We thus added an additional step for the other subsystems, we combined the two first averages which yielded the lowest *p* value and by multiplying the lowest one by 2 in order to give it more weight as compared to the second lowest. If no statistical significance was achieved, such as in the prosodic and timing subsystems, then average giving the lowest *p* value was kept as the feature assessing that subsystem. Different weighting factors were inspired by University of Michigan Classification of different dysarthria subtypes in APS^17^. We emphasize that we never used PD data in the design of the new features nor indices. PD data was used a posteriori as "controls" to potentially state the unrealibility of a particular new feature or index.

Using the scheme described above, for each subsystem task we ended up with the following features for each subsystem:

- Respiration subsystem:
  $${F}_{resp}=\frac{1}{2}\left(\frac{RL{R}_{m}+RL{R}_{t}+PI{R}_{m}+PI{R}_{t}}{4}+RS{R}_{m}+RS{R}_{t}\right)$$
  Hence, more weight/importance is given to *RSR* than *RLR* and *PIR* in the design of *F*_*resp*_, by a factor of 2.
- Phonation subsystem:
  $${F}_{phon}=\frac{1}{2}\left(\frac{Jitter+GV{I}_{m}+GV{I}_{t}}{3}+2{stdF0}_{a}\right)$$
  Hence, more weight/importance is given to *stdF*0_*a*_ than *Jitter* and *GVI* in the design of *F*_*phon*_, by a factor of 2.
- Articulation subsystem:
  $${F}_{art}=\frac{RF{A}_{m}+RF{A}_{t}+stdPSD}{3}$$
- Prosodic subsystem:
  $${F}_{pros}=\frac{{stdF0}_{m}+{stdF0}_{t}}{2}$$
- Timing subsystem:
  $${F}_{time}=\frac{ES{T}_{m}+RS{T}_{m}+AS{T}_{t}}{3}$$

These new features showed interesting behavior in term of statistical difference between groups, however they could not achieve a good classification accuracy, neither individually nor by a bivariate analysis where a classification was performed by considering a 2-dimensional input space with a feature in one dimension and another one in the second. We then grouped (and combined) the features in term of the class of impairment predominance. That is, the features reflecting an impairment which is more predominant in PSP (resp. MSA) are linearly combined by giving more weight to the feature showing the most significant impairment in PSP (resp. MSA). This led us to define 2 new speech subsystem indices (SSI) as:

- *SSI*_1_ as a combination of the 3 features *F*_*resp*_, *F*_*pros*_, *DUS* where we put more emphasize on the feature *F*_*resp*_:
  $${{{{{SSI}}}}}_{{{{\rm{1}}}}}={{{{{F}}}}}_{{{{{resp}}}}}+\frac{{{{{{DUS}}}}}_{{{{{m}}}}}}{{{{\rm{2}}}}}+\frac{{{{{{F}}}}}_{{{{{pros}}}}}}{{{{\rm{2}}}}},$$
- and *SSI*_2_ as a combination of the 3 features *F*_*art*_, *F*_*phon*_, *F*_*time*_ where we put more emphasize on the feature *F*_*art*_:
  $${{{{{SSI}}}}}_{{{{\rm{2}}}}}={{{{{F}}}}}_{{{{{art}}}}}+\frac{{{{{{F}}}}}_{{{{{phon}}}}}}{{{{\rm{2}}}}}+\frac{{{{{{F}}}}}_{{{{{time}}}}}}{{{{\rm{2}}}}}.$$

Note that while *DUS*_*m*_ and *F*_*art*_ both assess the articulation subsystem, they actually capture distinct articulation mechanisms.

Finally, we defined the composite speech subsystem index (CSSI) as the vector:

$${{{\rm{CSSI}}}}=({{{{{SSI}}}}}_{{{{\rm{1}}}}},{{{{{SSI}}}}}_{{{{\rm{2}}}}}).$$

### Design of acoustic indices by dysarthria type

After designing the 2 acoustic indices based on subsystem tasks, we used the findings to develop new indices which can be associated with subtypes of dysarthria. Tod do so, we first observed that *SSI*_1_ can be seen as an hypokinetic feature, we rename it then *H*1:

$$H1={SSI}_{1}={F}_{resp}+\frac{DU{S}_{m}}{2}+\frac{{F}_{pros}}{2}$$

As for *SSI*_2_, it is a combination of an hypokinetic feature *H*2 and an ataxic one *A*2:

$$H2={H}_{art}+\frac{{H}_{phon}}{2}+\frac{{H}_{time}}{2},$$

where *H*_*art*_, *H*_*phon*_ and *H*_*time*_ are hypokinetic articulation, phonation and timing features respectively: ${H}_{art}=\frac{{RFA}_{m}+{RFA}_{t}}{2}$, ${H}_{phon}=\frac{Jitter+{GVI}_{m}+{GVI}_{t}}{3}$, ${H}_{time}=\frac{{EST}_{m}+{RST}_{m}+{AST}_{t}}{3}$, and

$$A2=\frac{{stdF0}_{a}+stdPSD}{2}.$$

We then looked whether we could design other distinctive features related to particular dysarthria types. To do so, first we observe that *A*2 is obtained from the sustained phonation task only. On the other hand, it is known that syllables repetition is a fertile task to detect ataxic impairments. Following the same methodology as in subsystem feature design, we ended up defining an ataxic feature *A*1 which reflects a higher impairment in PSP:

$$A1=\frac{VD+DDKI}{2}.$$

Similarly, there is a relative consensus that PSP develop spastic dysarthria. We thus defined a spastic feature *S*1 as:

$$S1=\frac{1}{2}\left(\frac{NSR+DDKR}{2}+PSI\right).$$

This led us to define 2 dysarthria type indices (DTI) as:

$${{{{{DTI}}}}}_{{{{\rm{1}}}}}={{{{H1}}}}+\frac{{{{{A1}}}}+{{{{S1}}}}}{{{{{2}}}}}$$

and

$${{{{{DTI}}}}}_{{{{\rm{2}}}}}={{{{H2}}}}+{{{{A2}}}}.$$

Finally, we defined the composite dysarthria type index (CDTI) as the vector:

$${{{{CDTI}}}}=({{{{{DTI}}}}}_{{{{\rm{1}}}}},{{{{{DTI}}}}}_{{{{\rm{2}}}}}).$$

#### Statistical analysis

All analyses were performed in Python. The final speech values from the first and second run of sustained phonation, syllable repetition and reading task were averaged to provide greater stability of speech assessment^48^. The one-sample Kolmogorov–Smirnov test was used to evaluate the normality of distributions; the majority of acoustic features were found to be normally distributed. Group differences were calculated using analysis of variance for normally distributed data and the Kruskal–Wallis test for non-normally distributed data with the possible presence of outliers. A post hoc Tukey’s test was then applied to find differences between individual groups (HC vs. PD, HC vs. PSP, HC vs. MSA, PSP vs. MSA). Pearson and Spearman correlations were applied to test for significant relationships between normally and non-normally distributed data, respectively.

An overall indication of diagnostic accuracy was reported as the area under the curve (AUC), which we obtained from the receiver operating characteristic curve. The classification performance (sensitivity/specificity) of differentiating between groups was calculated using binary logistic regression with leave-one-speaker-out (LOSO) cross-validation. In PSP vs. MSA classification, the PSP group is considered as the positive label. In PD vs PSP/MSA classification, the PSP/MSA group is considered as the positive label.

## Supplementary information

Supplementary Material - List of audio files

Dataset 1

Dataset 2

Dataset 3

Dataset 4

## Data Availability

Individual participant data that underlie the findings of this study are available upon reasonable request from the corresponding author. The speech data are not publicly available due to their contain of information that could compromise the privacy of study participants. The analyses were performed using a publicly available Dysarthria Analyzer (Czech Technical University in Prague, available at http://dysan.cz/).
