# Supplementary material for: Speech acoustic indices for differential diagnosis between Parkinson’s disease, multiple system atrophy and progressive supranuclear palsy
Source: NPJ Parkinsons Dis. 2022 Oct 27;8:142. doi: 10.1038/s41531-022-00389-6 (PMC9613976; doi:10.1038/s41531-022-00389-6)
Supplement: Supplementary file 1 — Supplementary Material - List of audio files [file 41531_2022_389_MOESM1_ESM.pdf]

Supplementary Audio S1. Audio sample of sustained phonation, syllable repetition and reading passage of representative patient with progressive supranuclear palsy (Richardson syndrome, male, 69 years, and symptom duration 3 years).

Supplementary Audio S2. Audio sample of sustained phonation, syllable repetition and reading passage of representative patient with multiple system atrophy (parkinsonian subtype female, 67 years, and symptom duration 5 years).

Supplementary Audio S3. Audio sample of sustained phonation, syllable repetition and reading passage of representative patient with Parkinson's disease (male, 63 years, and symptom duration 5 years)

Supplementary Audio S4. Audio sample of sustained phonation, syllable repetition and reading passage of representative healthy control subject (male and 65 years).
